# Modular Flow Synthesis of Citric Acid-Coated Superparamagnetic Iron Oxide Nanoparticles: Preliminary Results

**DOI:** 10.3390/mi16111228

**Published:** 2025-10-29

**Authors:** Sabina Vohl, Andreja Nemet, Janja Stergar

**Affiliations:** Faculty of Chemistry and Chemical Engineering, University of Maribor, Smetanova 17, SI-2000 Maribor, Slovenia; sabina.vohl@um.si (S.V.); andreja.nemet@um.si (A.N.)

**Keywords:** superparamagnetic iron oxide nanoparticles, citric acid, modular flow microreactor system, continuous synthesis, zeta potential measurements

## Abstract

Superparamagnetic iron oxide nanoparticles (SPIONs) with sizes below 10 nm are biocompatible and non-toxic, making them promising for biomedical applications. To prevent their agglomeration and enhance their functionality, the nanoparticles were coated with citric acid (CA), which modifies the surface charge, improves dispersion stability, and facilitates biomedical use. In this work, a modular flow-through microreactor system was employed to synthesize and coat the nanoparticles in a single, continuous two-step process. The system enables precise control over temperature and mixing, ensuring uniform reaction conditions and minimizing hot spots. The synthesized Fe_3_O_4_ nanoparticles exhibited an average crystallite size of ~5 nm (XRD) and particle sizes of 4–6 nm (TEM). FTIR analysis confirmed the successful surface functionalization with CA, while TGA indicated a coating mass fraction of approximately 4–20 wt%, increasing with higher CA concentration. Zeta potential measurements revealed strong colloidal stability, with values around −35 mV at pH 6.5. Among the tested CA concentrations, the sample with a molar ratio of Fe_3_O_4_:CA = 1:0.25 exhibited the most favorable properties, including narrow size distribution and improved dispersion stability. These findings demonstrate that the continuous modular flow approach enables the reproducible synthesis of highly stable, sub-10 nm CA-coated SPIONs, offering promising potential for biomedical applications, particularly as magnetic resonance imaging (MRI) contrast agents.

## 1. Introduction

Superparamagnetic iron oxide nanoparticles (SPIONs) are typically defined as iron oxide nanoparticles with a core diameter below 10 nm, at which size thermal fluctuations are sufficient to randomize a magnetic moment, resulting in superparamagnetic behavior [1,2]. Intensive research over the past decades has demonstrated the broad applicability of SPIONs across diverse fields [3,4,5]. Studies on biocompatibility and toxicity have shown their suitability for biomedical application, particularly as contrast agents for magnetic resonance imaging (MRI) [6,7,8,9]. Furthermore, functionalization strategies enable the extension of their utilization to image-directed drug delivery. Their distinctive properties, such as high surface-area-to-volume ratio, magnetic properties that differ from their bulk counterparts, very high relaxivity, and extensive possibilities for surface functionalization with targeting molecules, have further advance their use in magnetic hyperthermia (MH) [3,10,11], magnetic separation [4,12], tissue engineering [3,13,14], and targeted delivery of drugs/therapeutic agents [9,15,16].

MRI remains one of the most reliable, non-invasive diagnostic techniques, capable of producing highly detailed anatomical images. In this technique, contrast agents are used to enhance the contrast between the target site and surrounding tissue [6,7]. Ferrite nanoparticles have proven to be effective contrast agents, as they modulate the nuclear spin relaxation process, thereby improving sensitivity and detection accuracy. Importantly, the performance of magnetic nanoparticles (MNPs) is strongly linked to their composition, size, surface properties, and degree of agglomeration in the biological environment [4,8]. Therefore, understanding the relationship between the intrinsic parameters and the relaxivities of nuclei influenced by MNPs can provide crucial insights for accurately predicting the properties of engineered MNPs and improving their performance in MRI-based theranostic applications [17].

The initial step in developing SPIONs is the synthesis of the magnetic core. Numerous methods have been proposed to achieve precise control over the size, shape, and magnetic properties. A highly reproducible synthesis process is crucial, as this is one of the essential requirements for the potential clinical translation of these nanomaterials [4]. Generally, nanoparticles smaller than 15 nm are preferred. At these sizes, ferri- and ferromagnetic nanoparticles achieve a single magnetic domain, characterized by a large magnetic moment. Such particles exhibit superparamagnetic properties, including high magnetic moments and rapid response to external magnetic fields, while showing negligible remanent magnetization and coercivity when the external magnetic field is removed [18,19]. This ensures that SPIONs lose their magnetization immediately after the magnetic field is removed, preventing unwanted nanoparticle aggregation and ensuring safe removal from the body. However, due to their small size and high surface-to-volume ratio, SPIONs tend to aggregate as a result of their high surface energy [6,19,20]. Uncoated iron oxide particles are unstable and susceptible to oxidation in the air, which reduces magnetic properties. To maintain and enhance their long-term stability in physiological environments, MNPs must be appropriately surface-modified or coated. The coating material also determines the final surface charge of the MNPs [3,6]. Various synthesis methods have been developed to produce SPIONs with the desired size, shape, saturation magnetization, and other key properties. These methods can be either one-step or two-step, particularly when additional functionalization is required, such as protective coating or surface functionalization aimed at specific applications [6,19,21,22].

Carboxylates greatly influence the development and magnetic properties of SPIONs. For example, surface modification with heavy-chain fatty acids or thiols enhances stability of aqueous SPION suspensions [23]. Coating nanoparticles with citric acid (CA) results in a thermodynamically stable colloidal solution. CA is a widely used organic coating material because it can modify surface charge and hydrophobicity while leaving free carboxyl groups available on the nanoparticle surface. In addition to its stabilizing role, CA exhibits bactericidal and bacteriostatic properties, and solutions containing CA are commonly used as stabilizing agents and plant growth stimulators. Importantly, nanosystems composed of water-dispersed SPIONs coated with CA demonstrate high biocompatibility and low toxicity. This makes them particularly valuable in biomedical applications, where CA acts as a linker for anticancer drugs and enhances the use of SPIONs as drug delivery systems and MRI contrast agents [23,24,25].

In this work, a modular flow-through microreactor system was used to synthesize and coat nanoparticles within a single, continuous process setup using two different microreactors. This change from batch production to semi-continuous flow chemistry offers several key advantages [26,27]. First, the precise control of reaction parameters minimizes temperature and concentration gradients, thereby reducing the risk of hot spots. In batch systems, mixing typically occurs by diffusion over several seconds, which often results in broad particle size distributions (standard deviation > 20%) and increased aggregation. In contrast, microreactors provide characteristic mixing times below 100 ms due to their high surface-to-volume ratios and efficient mass and heat transfer, leading to narrower particle size distributions (standard deviation < 5–10%) and enhanced control over nucleation and growth dynamics [28,29]. Second, the inherently enhanced heat and mass transfer in microreactors significantly accelerates reaction kinetics compared to batch processes. Third, microreactors offer excellent scalability and reproducibility; scale-up can be achieved through the parallelization of identical units without altering the optimized reaction environment, ensuring consistent product quality across production scales. These quantitative advantages are especially beneficial for achieving homogeneous citric acid coating and stable colloidal dispersions of magnetic nanoparticles, as rapid and uniform mixing minimizes local concentration and pH gradients that could otherwise compromise coating efficiency and long-term stability [30]. Moreover, the modular design of the microreactor system employed in this study aligns conceptually with recently reported “modular structure” strategies in nanomaterial synthesis, which emphasize design flexibility, process control, and adaptability to diverse reaction schemes [31].

In this work, we present a modular flow-through microreactor system for the continuous synthesis and in situ CA coating of SPIONs within a single, integrated process. This approach allows precise control over reaction parameters, enabling the production of nanoparticles with uniform size and morphology. Among various biomedical applications of SPIONs, MRI is particularly sensitive to nanoparticle size and magnetic properties. Particles smaller than 10 nm exhibit strong superparamagnetic behavior, high transverse relaxivity, and excellent colloidal stability, making them ideal T_2_ contrast agents. The SPIONs synthesized in this study, with an average size of approximately 5 nm, therefore possess characteristics that are especially favorable for MRI-based applications.

The novelty of this work lies in the integration of continuous flow synthesis and in situ surface functionalization with CA within a modular microreactor platform. This combined, sequential, and continuous approach offers enhanced control over particle size, dispersion, and surface stability compared to conventional batch or multi-step coating methods, representing an important step toward scalable and reproducible production of CA-coated SPIONs for biomedical use.

## 2. Materials and Methods

### 2.1. Materials

− Iron(II) chloride, tetrahydrate, 98%,
− Iron(III) chloride,
− Sodium hydroxide,
− Hydochloric acid, 37%,
− CA,
− Purified water,
− Absolute ethanol (CH_3_CH_2_OH).

All the chemicals needed were purchased from Sigma Aldrich. Purified water was used for preparing solutions, and purified water and absolute ethanol were used for washing MNPs.

Three solutions were prepared for this synthesis.

(i) 3 M NaOH solution,
(ii) Iron salt precursor solution (ISPS) that contained 366 mg FeCl_2_·4H_2_O and 566 mg FeCl_3_ in 36 mL 1 M HCl,
(iii) 1 M CA.

### 2.2. Modular Flow Microreactor System Setup

The synthesis and coating of nanoparticles were performed using a modular Asia Syrris flow microreactor system, where the nanoparticle synthesis follows a demonstration case of Asia Syrris, while the upgrading process coats them with citric acid, consisting of modules as follows:

− Asia pump,
− Asia Automated RIM,
− Chip reactor 250 µL and 1 mL,
− Back pressure controller,
− Automated collector.

**Figure 1:**
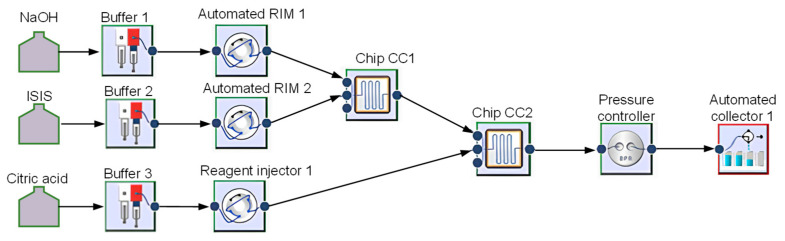
Schematic setup of the simultaneous production of nanoparticles and coating with CA.

Purified water served as the carrier liquid in the system. The first (Buffer 1 in Figure 1) and second channel (Buffer 2 in Figure 1) of the first pump were connected to the first channel (Automated RIM 1 in Figure 1) and second channel (Automated RIM 2 in Figure 1) of the automated reagent injection system, enabling automated dosage reagents. The 3 M NaOH solution and ISPS were introduced into 250 µL (Chip (CC) 1 in Figure 1) at 90 °C with residence time 0.25 min (15 s), where they reacted to form magnetite/maghemite nanoparticles according to Equation (1).

2 Fe^3+^_(aq)_ + Fe^2+^_(aq)_ + 8 Cl^−^_(aq)_ + 8 Na^+^_(aq)_ + 8 OH^−^_(aq)_ → Fe_3_O_4(s)_ + 8 NaCl_(aq)_ + 4 H_2_O_(l)_, (1)

The nanoparticle suspension was transferred into a second 1 mL (Chip CC(2) in Figure 1), where it was mixed with 1 M CA, supplied by the manual reagent injection system (Reagent Injector 1 in Figure 1) using the first channel of the second pump (Buffer 3 in Figure 1) to provide continuous flow. For all three reagent injection systems the 5 mL loop was used, which then presented the limiting factor in the quantity of product that can be produced in one run. In the second microreactor, the following reactions take place. CA dissociates in aqueous solution (Equation (2)) and the citrate ions coordinate with Fe_3_O_4_ nanoparticles via their carboxylate groups (-COO^−^) as simplistically presented in Equation (3). By these reactions, a formation of stable Fe_3_O_4_@CA coating is obtained. The residence time in the second reactor was 1 min at a temperature of 80 °C.

C_6_H_8_O_7(aq)_ → C_6_H_5_O_7_^3−^_(aq)_ + 3 H^+^_(aq)_, (2)

Fe_3_O_4(s, nanoparticles)_ + C_6_H_5_O_7_^3−^_(aq)_ → Fe_3_O_4_@CA_(coated nanoparticles)_, (3)

Direct pH monitoring within the microreactor was not feasible, as conventional pH probes require larger sample volumes and exhibit response times longer than the residence times in the flow channels. However, due to the precise and reproducible control of flow and mixing provided by the Asia Syrris microreactor system, and based on validation experiments, it can be assumed that the local pH conditions remained consistent for all synthesis runs.

The overall net reaction together in both reactions is presented in Equation (4).

FeCl_2_·4H_2_O_(aq)_ + 2 FeCl_3(aq)_+ 8 NaOH_(aq)_+ C_6_H_8_O_7(aq)_→ Fe_3_O_4_@CA_(s)_+ 8 NaCl_(aq)_ + 13 H_2_O_(l)_, (4)

NaCl and water do not participate in the coating process, but they can influence the ionic strength and pH of the solution that have an impact on the coating efficiency and stability. The common pH range for the coating process is between 4 and 7. The citrate coating provides a negative charge to the surface of MNPs, obtaining electrostatic repulsion between particles and preventing aggregation of coated nanoparticles. It should be noted that the amount of added CA can greatly affect the products, as instead of coating the nanoparticles the iron oxide can be dissolved when pH is below 4.

The process was maintained at 1 bar with the backpressure controller (Pressure Controller 1 in Figure 1) at 1 bar of pressure, and the coated nanoparticles in suspension were collected with the automated collector (Automated Collector 1 in Figure 1).

### 2.3. Experimental Setup

Different molar ratios of CA to nanoparticles were tested to optimize coating efficiency. The samples are denoted as MNP@CA*x*, where *x* represents the molar ratio of citric acid to Fe_3_O_4_ (Table 1).

### 2.4. Analysis/Characterization of Fe_3_O_4_@CA

To confirm the successful coating of magnetic MNP with CA, the average hydrodynamic particle size, zeta potential, and isoelectric point (IEP) were determined by dynamic light scattering (DLS, Zetasizer Nano ZS, Malvern, Worcestershire, UK) using purified MNP@CA water dispersions. Measurements were conducted with purified water, and the pH was adjusted from the current pH value to 2.0 with 0.1 M hydrochloric acid (HCl) using an auto titrator.

The functional groups of MNP@CA were analyzed using Fourier transform infrared spectroscopy (FTIR, IRAffinity-1S, Shimadzu, Tokyo, Japan). The FTIR spectrum was measured in the range of 4000–400 cm^−1^. The size and morphology of the nanoparticles were determined using transmission electron microscopy (TEM, JEM-2010F, JEOL, Tokyo, Japan).

The empirical size distribution of the MNPs was estimated by measuring the area of the MNPs on the TEM image. The average particle size is given as a number-weighted average equivalent diameter, representing the diameter of a circle with the same area as the imaged particle.

Phase identification and core particle characterization were performed by X-ray powder diffraction (XRD, high-resolution X-ray powder diffractometer PANalytical X’Pert PRO MPD with Cu–Kα_1_ radiation (λ = 1.5406 Å)).

The weight loss of Fe_3_O_4_@CA was characterized by thermogravimetric analysis (TGA, TGA2, Mettler Toledo, Greifensee, Switzerland). The measurement was performed under nitrogen (100 mL/min) from 25 °C to 600 °C at a heating rate of 10 K/min.

## 3. Results and Discussion

### 3.1. X-Ray Powder Diffraction

**Figure 2:**
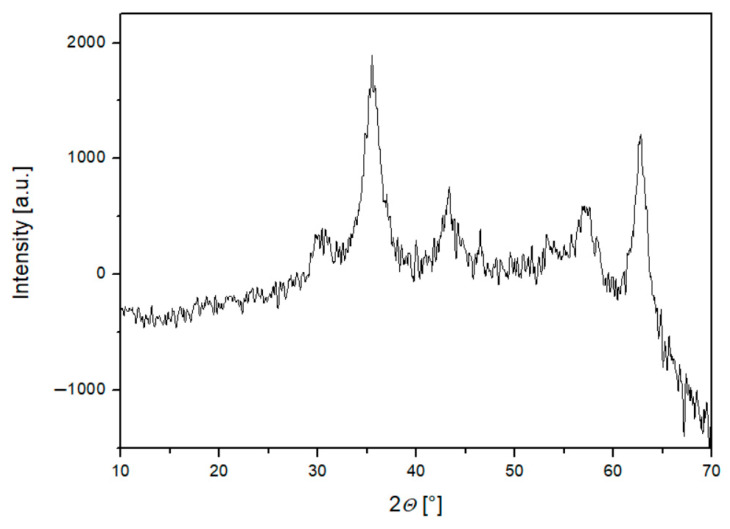
XRD spectra of Fe_3_O_4_.

Based on the diffraction data of the obtained samples, the diffraction peaks at 2*Θ* values were assigned to the crystal planes (220), (311), (400), (511), and (440), respectively (Figure 2). For Fe_3_O_4_, peaks were observed at 30.2°, 35.6°, 43.2°, 53.4°, 57.2°, and 62.7° at 2*Θ* degrees, indicating the cubic spinel structure of magnetite [32]. All peaks correspond well with the characteristic peaks of magnetite (JCPDS file no. 00-019-0629). The average crystallite diameter, *d*_x_ = 5 nm, was calculated using the Scherrer equation. The average value of the lattice parameter was determined to be *a* = 0.8358 nm. Similar peaks were found for Fe_3_O_4_@CA, revealing that the CA coating does not cause a phase change in the bare Fe_3_O_4_ [33]. The average crystallite size, estimated using the Scherrer equation, was approximately 5 nm. This value serves as an approximate indicator of particle size, as effects such as lattice strain or instrumental broadening were not separately analyzed. The obtained size is consistent with TEM observations (4–6 nm), confirming the nanoscale dimensions of the synthesized Fe_3_O_4_ particles.

### 3.2. Fourier Transform Infrared Spectroscopy

**Figure 3:**
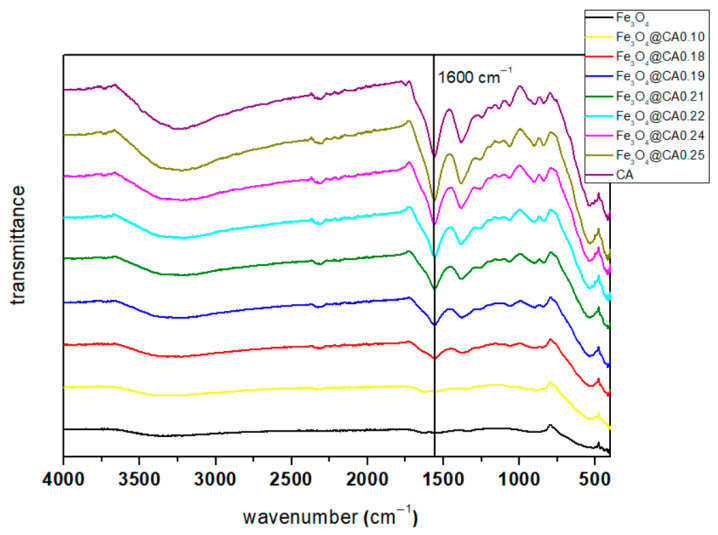
FTIR spectra of Fe_3_O_4_, Fe_3_O_4_@CA.

The FTIR spectrum range (4000–400 cm^−1^) of Fe_3_O_4_@CA corresponding to the iron citrate spectrum is presented. A large and intense band at 3450 cm^−1^ can be attributed to the structural OH groups as well as traces of molecular water and CA. The peak at 1700 cm^−1^, assignable to the C=O vibration (symmetric stretching) from the COOH group of CA, shifts to an intense band at about 1600 cm^−1^ for Fe_3_O_4_ coated with CA (Fe_3_O_4_@CA), indicating the binding of a CA radical to the magnetite surface, as shown in Figure 3. The band at 1400 cm^−1^ can be assigned to the asymmetric stretching of CO from the COOH group. The low-intensity bands between 400 and 600 cm^−1^ can be associated with the stretching and torsional vibration modes of the magnetite. These assignments are consistent with a previous study [34] that described two broad bands at 580 and 400 cm^−1^ associated with magnetite (Fe_3_O_4_). As can be seen from Figure 3, coating with CA was successful at some rates for most of the mass ratio of MNP and CA. The depth of the transmittance suggests that a higher amount of CA leads to more coated SPIONs. Only for sample Fe_3_O_4_@CA0.10 can it be concluded that coating with CA was not successful.

### 3.3. Thermogravimetric Analysis

**Figure 4:**
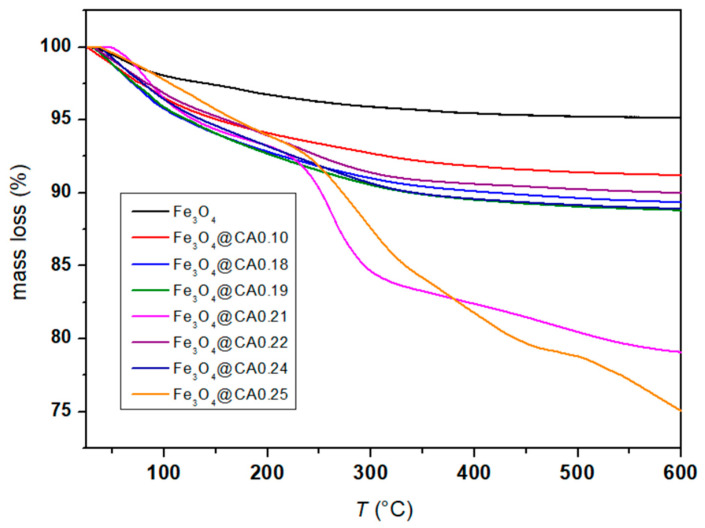
TGA of the Fe_3_O_4_ and Fe_3_O_4_@CA.

A TGA was conducted to measure the organic content of Fe_3_O_4_@CA. The TGA results are presented in Figure 4, which illustrates the mass loss of Fe_3_O_4_@CA with different molar ratios between Fe_3_O_4_ and CA. A correction of 3% due to the oxidation of magnetite to hematite was taken into account, and the mass loss of CA was accordingly adjusted. The mass loss curve observed between 100 and 600 °C is attributed to the evaporation of water and the decomposition of the bound citrate molecules on the Fe_3_O_4_ MNP surfaces. The initial mass loss below 160 °C was due to the evaporation of physically adsorbed water, and the rest of the mass loss was determined as organic content of the coated Fe_3_O_4_@CA. This represents the mass percentage of all surface-coated organic groups on the MNPs, corresponding to the desorption of CA molecules from the magnetite particles. The mass percentage loss of CA of each sample is presented in Table 2. The formation of a CA coating layer on the particle surface is attributed to the chemical bonding between the carboxyl groups of CA and the Fe-OH sites on the iron oxide nanoparticles [35].

The amount of organic shell on the surface of nanoparticles was evaluated with thermogravimetry. Based on the graph (Figure 4) and Table 2, it can be concluded that CA constitutes between 4% and 20% of the sample mass [25].

### 3.4. Transmission Electron Microscopy

**Figure 5:**
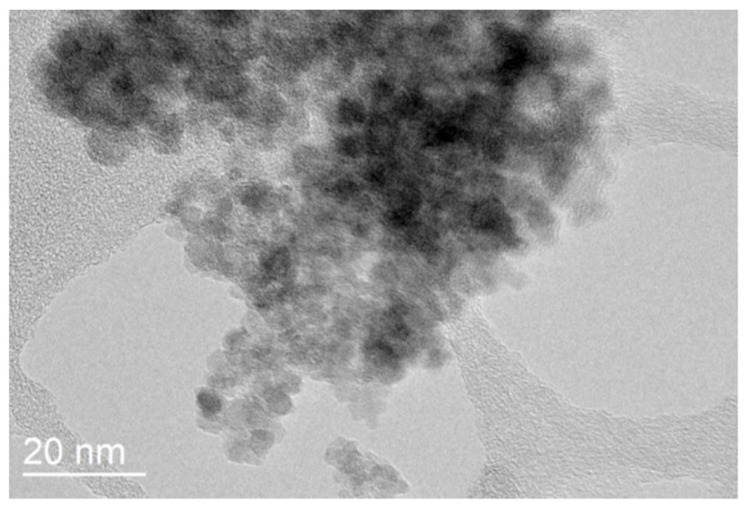
TEM image of bare Fe_3_O_4_ nanoparticles.

Figure 5 shows that the monodispersed rough surface of Fe_3_O_4_ was confirmed through TEM analysis, with particle sizes in the range of 4–6 nm. These values are consistent with the XRD results, where the crystallite size was estimated to be around 5 nm. The crystallite size calculated from XRD is in good agreement with the particle size observed in TEM images. Minor deviations are expected, as XRD provides the size of coherent crystalline domains, while TEM measures the overall particle size, which may include surface coatings or slight aggregation. In comparison with the literature, our findings demonstrate that the microreactor-based flow synthesis yields exceptionally small MNPs relative to those obtained by conventional synthesis methods [36]. For example, Boosz et al. [37] reported a Z-average size of approximately 10 nm for SPIONs synthesized via batch processes, which is notably larger than the particles produced in our study. Additionally, batch synthesis can lead to less uniform coating and stability issues, which are mitigated in our continuous flow approach, highlighting the advantages of modular microreactor-based synthesis for producing ultrasmall, well-dispersed nanoparticles.

### 3.5. Zeta Potential Measurements

An effective way to enhance the water stability of nanoparticles is by altering their isoelectric point (IEP) through surface modification. In this study, CA was used to coat the Fe_3_O_4_ nanoparticles. Citrate, with its three carboxyl groups, served as a potent ligand for stabilizing Fe_3_O_4_ particles [38]. The surface charges of the bare magnetite nanoparticles and Fe_3_O_4_@CA were characterized by measuring their zeta potential as a function of pH ranging from 7.0 to 2.0, with each measurement conducted in triplicate. Dispersion stability is related to the zeta potential value (mV): a range of 0 to ±5 can lead to rapid agglomeration and precipitation of nanoparticle suspensions; ±10 to ±30 marks the threshold for delicate dispersion; ±30 to ±40 indicates moderate stability of colloidal nanoparticles; and ±40 to ±60 signifies excellent stability of nanoparticle suspensions, indicating a high charge on their surface [23].

**Figure 6:**
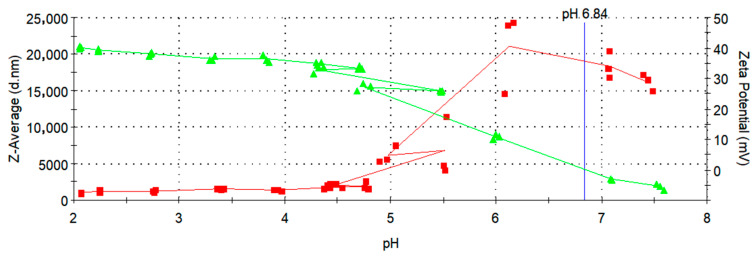
Change in zeta potential and Z-average for Fe_3_O_4_ with pH values.

**Figure 7:**
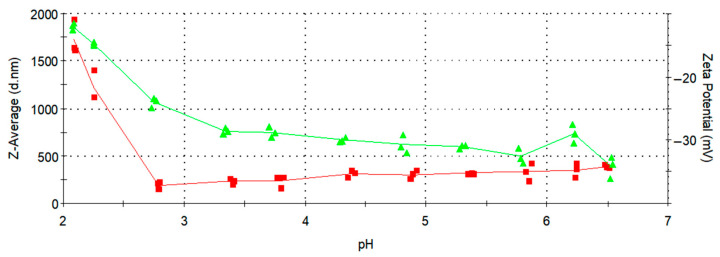
Change in zeta potential and Z-average for Fe_3_O_4_@CA0.25 with pH values.

In Figure 6 and Figure 7, the zeta potential values are indicated by green triangles and a green line. As shown in Figure 6, the bare MNPs exhibits an IEP at pH 6.84, consistent with literature reports for Fe_3_O_4_ [36], whereas the Fe_3_O_4_@CA displays a negative zeta potential of −35 mV at pH 6.5 (Figure 7), respectively. This observed behavior is likely due to the adsorption of citrate onto the bare MNPs, altering the surface charges because of the presence of surface-bound carboxylate groups. The zeta potential becomes more negative with increasing pH due to the rise in OH^−^ ions in the solution and the deprotonation of the carboxyl groups of CA, given that the pKa values of citrate are 3.1, 4.8, and 6.4, respectively [36].

This confirms the presence of negatively charged carboxylate groups on the surface of the MNPs. The resulting electrostatic repulsion ensures their colloidal stability in aqueous suspension, as some of the carboxylate groups from CA are adsorbed or coordinated on the MNPs’ surface, while uncoordinated species extend into the water medium [39]. Consequently, the effective charge at pH 6,5 provides stabilization of Fe_3_O_4_@CA MNPs. The effective hydrodynamic diameter of Fe_3_O_4_@CA MNPs, measured by DLS at 25 °C, varies from 188.7 to 1730 nm with changing pH. The effective particle size started to increase at low pH values below 3, as in this range (pH 3 to 2) the zeta potential markedly decreased and approached zero. As shown in Figure 7, the IEP was not reached within the measured interval, since its value lies below pH 2, while our measurements were conducted in the range from pH 7 to 2.

Based on the results shown in Figure 6 and Figure 7, the IEP differs substantially between bare Fe_3_O_4_ nanoparticles, with an IEP of 6.84, and Fe_3_O_4_@CA0.25, where the IEP is below 2. This indicates that the nanoparticles were successfully coated with CA, as a surface modification has clearly occurred. Our study reports an average particle size of approximately 5 nm and a zeta potential of around −35 mV for the CA-coated SPIONs synthesized using a modular flow microreactor system. These values are consistent with those reported in previous studies employing similar synthesis or surface modification strategies. For instance, Hussein et al. [40] observed comparable zeta potential values and enhanced colloidal stability for CA-coated SPIONs prepared via chemical co-precipitation, confirming the effectiveness of citric acid in providing electrostatic stabilization. This agreement further validates that the microreactor-based synthesis approach yields nanoparticles with physicochemical properties suitable for biomedical applications.

The modular flow microreactor system offers several advantages over traditional batch methods, including precise control over reaction parameters such as temperature, mixing, and reagent flow, which results in more uniform reaction conditions and consistent particle sizes. This precise control minimizes temperature and concentration gradients, thereby improving reproducibility and colloidal stability of the synthesized nanoparticles. Moreover, the continuous nature of the process allows for facile scalability through parallelization of identical units without altering the optimized conditions.

These benefits are consistent with recent studies that emphasize the advantages of continuous flow synthesis in achieving improved size uniformity, stability, and production efficiency of nanomaterials. Estévez et al. [41] similarly demonstrated that continuous flow microreactor systems enhance nanoparticle quality and process reproducibility compared to conventional batch methods, underscoring the potential of this approach for scalable and controlled nanoparticle production.

## 4. Conclusions

In this study, SPIONs were successfully synthesized and simultaneously coated with CA using a modular flow microreactor system. This integrated approach enabled continuous, well-controlled synthesis, combining nucleation, growth, and surface functionalization in a single process. XRD analysis confirmed the formation of Fe_3_O_4_ nanoparticles with an average crystallite size of approximately 5 nm, which was consistent with the TEM observations showing nearly spherical particles with sizes in the range of 4–6 nm. FTIR spectra verified the effective surface coating with CA, while TGA analysis demonstrated increased mass loss with higher CA concentrations, confirming successful coating. Zeta potential measurements around −35 mV at pH 6.5 further indicated strong colloidal stability of the coated particles.

Compared to conventional batch synthesis methods reported in the literature, the microreactor-based approach provides enhanced control over reaction parameters and reproducible particle characteristics within the sub-10 nm range. The combination of small particle size, narrow size distribution, and good dispersion confirms the potential of this continuous flow process for scalable nanoparticle production.

Overall, this study presents preliminary results demonstrating the feasibility and promise of modular flow synthesis for producing CA-coated SPIONs. Future work will focus on quantitative scalability assessment, process reproducibility, and optimization of coating uniformity to further advance the application of this approach for biomedical purposes, particularly as MRI contrast agents and drug delivery platforms.

Future work will focus on improving experimental reproducibility through replicate synthesis and statistical evaluation, as well as on detailed characterization of coating uniformity using advanced techniques. Since direct pH monitoring within the microreactor was not feasible in the present setup, the development of integrated micro-scale pH sensing or feedback control will be pursued to further enhance coating precision and process consistency. In addition, the scalability of the modular microreactor system will be examined through extended operation and parallelization studies to ensure stable and uniform nanoparticle production.

## Figures and Tables

**Table 1:** Sample names and corresponding molar values of MNPs and CA used in this study.

Name of Sample	Amount of MNPs [mol]	Amount of CA [mol]
Fe_3_O_4_@CA0.10	1	0.10
Fe_3_O_4_@CA0.18	1	0.18
Fe_3_O_4_@CA0.19	1	0.19
Fe_3_O_4_@CA0.21	1	0.21
Fe_3_O_4_@CA0.22	1	0.22
Fe_3_O_4_@CA0.24	1	0.24
Fe_3_O_4_@CA0.25	1	0.25

**Table 2:** Mass loss of CA for different molar ratios of Fe_3_O_4_@CA.

Sample	Mass Loss of CA (%)
Fe_3_O_4_@CA0.18	4.4
Fe_3_O_4_@CA0.19	4.9
Fe_3_O_4_@CA0.21	5.0
Fe_3_O_4_@CA0.22	5.3
Fe_3_O_4_@CA0.24	15.0
Fe_3_O_4_@CA0.25	19.6

## Data Availability

The data presented in this study are available on request from the corresponding author.

## Abbreviations

The following abbreviations are used in this manuscript:

CA	Citric acid
DLS	Dynamic light scattering
FTIR	Fourier transform infrared spectroscopy
IEP	Isoeletric point
ISPS	Iron salt precursor solution
MH	Magnetic hyperthermia
MNPs	Magnetic nanoparticles
MRI	Magnetic resonance imaging
SPION	Superparamagnetic iron oxide nanoparticles
TEM	Transmission electron microscopy
TGA	Thermogravimetric analysis
XRD	X-ray powder diffraction
